# PPAR-Alpha Agonists as Novel Antiepileptic Drugs: Preclinical Findings

**DOI:** 10.1371/journal.pone.0064541

**Published:** 2013-05-27

**Authors:** Monica Puligheddu, Giuliano Pillolla, Miriam Melis, Salvatore Lecca, Francesco Marrosu, Maria Graziella De Montis, Simona Scheggi, Gianfranca Carta, Elisabetta Murru, Sonia Aroni, Anna Lisa Muntoni, Marco Pistis

**Affiliations:** 1 Department of Public Health, Clinical and Molecular Medicine, University of Cagliari, Cagliari, Italy; 2 Department of Biomedical Sciences, University of Cagliari, Cagliari, Italy; 3 Department of Molecular and Developmental Medicine, University of Siena, Siena, Italy; 4 C.N.R. Neuroscience Institute, Cagliari, Italy; University Paris 6, France

## Abstract

Nicotinic acetylcholine receptors (nAChRs) are involved in seizure mechanisms. Hence, nocturnal frontal lobe epilepsy was the first idiopathic epilepsy linked with specific mutations in α4 or β2 nAChR subunit genes. These mutations confer gain of function to nAChRs by increasing sensitivity toward acetylcholine. Consistently, nicotine elicits seizures through nAChRs and mimics the excessive nAChR activation observed in animal models of the disease. Treatments aimed at reducing nicotinic inputs are sought as therapies for epilepsies where these receptors contribute to neuronal excitation and synchronization. Previous studies demonstrated that peroxisome proliferator-activated receptors-α (PPARα), nuclear receptor transcription factors, suppress nicotine-induced behavioral and electrophysiological effects by modulating nAChRs containing β2 subunits. On these bases, we tested whether PPARα agonists were protective against nicotine-induced seizures. To this aim we utilized behavioral and electroencephalographic (EEG) experiments in C57BL/J6 mice and *in vitro* patch clamp recordings from mice and rats. Convulsive doses of nicotine evoked severe seizures and bursts of spike-waves discharges in ∼100% of mice. A single dose of the synthetic PPARα agonist WY14643 (WY, 80 mg/kg, i.p.) or chronic administration of fenofibrate, clinically available for lipid metabolism disorders, in the diet (0.2%) for 14 days significantly reduced or abolished behavioral and EEG expressions of nicotine-induced seizures. Acute WY effects were reverted by the PPARα antagonist MK886 (3 mg/kg, i.p.). Since neocortical networks are crucial in the generation of ictal activity and synchrony, we performed patch clamp recordings of spontaneous inhibitory postsynaptic currents (sIPSCs) from frontal cortex layer II/III pyramidal neurons. We found that both acute and chronic treatment with PPARα agonists abolished nicotine-induced sIPSC increases. PPARα within the CNS are key regulators of neuronal activity through modulation of nAChRs. These effects might be therapeutically exploited for idiopathic or genetically determined forms of epilepsy where nAChRs play a major role.

## Introduction

Binding of nicotine to nicotinic acetylcholine receptors (nAChRs) elicits in laboratory animals dose-dependent effects that begin with hypermotility and culminate with clonic-tonic seizures and death at high doses [1]. Investigation on the mechanisms underlying nicotine-induced seizures might help to understand how nAChRs participate in the mechanisms of epilepsy. Interest for nAChR in several epileptic syndromes previously considered “idiopathic” was rekindled by the finding that altered functional properties of nAChR are implicated in the pathogenesis of nocturnal frontal lobe epilepsy (NFLE), and that seizures induced by nicotine in rodents model nAChR-related epilepsy. NFLE is linked with mutations of the α4 or β2 subunits [2], [3], [4], [5], [6], [7], the most abundantly expressed subunits in the CNS [8]. Though in human NFLE, or in genetically engineered mice that model the disease, functional properties of nAChRs are intimately altered, nicotine evokes seizures by over-activating a healthy system. However, several neurophysiological events ultimately leading to seizures may share common steps between these conditions. Indeed, the common trait in enhancing the epileptogenesis is the over-activation of cholinergic systems, either pharmacologically induced or mediated by the gain of function that mutated nAChRs exhibit toward their ligands [9]. These effects might be based on the extensive expression of nAChRs, particularly those containing the β2 subunit (β2*nAChRs) in thalamo-cortical, hippocampal and frontal regions [10]. Indeed, altered cholinergic activation of neocortical and/or thalamocortical networks plays a central role in the generation of both nicotine-induced and NFLE seizures, which originate in the frontal cortex (FCx) [11] and specifically affect GABA_A_-mediated inhibitory inputs to pyramidal neurons [12].

Based on this evidence, negative regulation of nAChRs might represent a potential therapeutic approach in nAChR-related forms of epilepsies.

We previously discovered that nicotine-evoked excitation of dopamine neurons both *in vivo* and *in vitro*, as well as nicotine addictive properties in rats and monkeys, are suppressed by ligands to the peroxisome-proliferator-activated receptor-α (PPARα) [13], [14], [15], [16]. PPARα is one of three subtypes of the nuclear receptor PPAR family [17], [18], [19], expressed by neurons in many brain regions [20], [21], and activated by endogenous ligands, the N-acylethanolamines oleoylethanolamide (OEA) and palmitoylethanolamide (PEA) [22], and by synthetic ligands such as hypolipidemic fibrates [16].

Evidence points to a non-transcriptional interaction between PPARα and nAChR, via phosphorylation [13], [14], [23]. This mechanism might account for the blockade of neuronal and behavioral responses to nicotine [13], [15], [16].

Building upon these findings, we postulated that PPARα agonists might display anticonvulsant properties. Thus, we first investigated whether acutely or chronically administered PPARα ligands reduce the severity of nicotine-induced seizures. Next, we assessed whether these compounds regulate the phosphorylation status of the β subunit of the nAChR and abolish nicotine-induced enhancement of inhibitory currents on pyramidal neurons in layers II/III of the frontal cortex (FCx) in mice and rats.

## Materials and Methods

### Animals

For behavioral experiments, electroencephalographic (EEG) and patch-clamp recordings male C57BL/6J mice (Harlan, San Pietro al Natisone, Italy) (*n* = 80; weight: 25–30 g each) were used. For immuno-blotting experiments and patch-clamp recordings, we utilized male Sprague-Dawley rats (250–300 g and 14–21 d, respectively, n = 30, Harlan, San Pietro al Natisone, Italy) Mice and rats were housed six per cage under a 12 h light/dark cycle (light on at 7∶00 AM), in conditions of constant temperature (21±2°C) and humidity (60%), with food and water *ad libitum*.

### Ethics Statement

Experiments were performed in strict accordance with the EEC Council Directive of 24 November 1986 (86/609). All efforts were made to minimize pain and suffering and to reduce the number of animals used. The experimental protocols were also approved by the Animal Ethics Committee of the University of Cagliari.

### Treatments

Animals were randomly assigned to the experimental groups undergoing behavioral seizure scoring or EEG recordings. In the first set of experiments a dose-response curve for nicotine (5, 7, 10 mg/kg) was carried out. Each mouse received a single dose of nicotine.

Subsequent acute experiments, aimed to assess the effect of the PPARα agonist WY14643 or the antagonist MK886, were carried out with the 10 mg/kg dose of nicotine. In these cases, mice received two injections, spaced by 10 min intervals, before the final nicotine administration after 15 min: we first injected the antagonist MK886 (3.0 mg/kg, i.p.) or its vehicle followed by the administration of WY14643 (80 mg/kg, i.p.) or its vehicle.

For chronic studies, one week after EEG electrode implants (see below) mice were divided into diet treatment groups: (i) a standard diet (control group, Harlan Teklad Global 2016); (ii) a 0.2% fenofibrate diet (Fenofibrate from Sigma-Aldrich+Harlan Teklad Global 2016). Mice were fed diets for 14 days. On the day of the experiment, mice received one injection [MK886 (3.0 mg/kg, i.p.)] or its vehicle before nicotine (10 mg/kg) administration after 15 min.

### Seizure Scoring

Immediately after nicotine injection, mice were placed in a regular mouse cage with bedding, and behavioral responses were recorded for 5 min. The symptoms were scored independently by two experimenters blind to the treatment on an arbitrary scale from 0 to 6 (modified from Franceschini et al., 2002) [24] as follows: 0, no visible effects; 1, locomotor effects including increased exploring activity and/or sedation; 2, tachypnea, tremors, back arching; 3, any combination of the symptoms in 1 and 2 plus rapid movements of the legs, wild running, or partial loss of righting reflex; 4, any combination of the previous symptoms plus complete loss of righting reflex, clonic seizures, 5, any combination of the preceding symptoms plus tonic seizures; 6, death, with or without hyperextension of the limbs along the axis of the body (soldier position).

### EEG Recordings

Mice were anesthetized with Equithesin (5 ml/kg, i.p.) and placed in a stereotaxic apparatus (David Kopf, mod. 900). The skull was exposed and perforated, the holes aimed at the following positions: one located above the (either left or right) sensorimotor cortices (FPr and FPl), [coordinates from bregma (mm): anteroposterior +2, lateral 3, ventral to skull surface 1.5], two targeted to the dorsal hippocampus with bipolar leads glued together [coordinates from bregma (mm): anteroposterior +2, lateral 1 and 2, ventral 1.3 and 2] and one on the skull over the cerebellum as a reference [coordinates from bregma (mm): anteroposterior −5.9, lateral 1.5] [25]. A four-pin male socket was positioned into the holes, secured to the skull with epoxy resin and covered with acrylic cement to improve retention.

One week following surgical preparation of the animals (or three to five weeks for chronic studies), experiments started according to the protocol described above, electrical potentials were acquired and the signals amplified, bandpass-filtered and recorded on a portable digital EEG polygraph (BQS 98 System Micromed). In addition, in view of the possibility that digital data generated by the above mentioned experimental settings may need unconventional processing, further home-made software analysis rewritten on a Matlab® platform was used (e.g. signal analysis with wavelets versus traditional fast Fourier transform (FFT) processing).

Electrode impedance was maintained at <5 kΩ. The amplified signals, processed with a band-pass filter (0.02 to 70 Hz), was stored on the hard disk at a sampling rate of 256/s.

The behavior of the animals was video-recorded for the entire duration of the experiment.

### In vitro Electrophysiology

The preparation of FCx slices and whole-cell patch clamp recordings from layer II/III pyramidal neurons was as described previously [12]. Briefly, male mice (10–25 d, Harlan) and Sprague Dawley rats (14–21 d, Harlan) were anesthetized with halothane and killed. A block of tissue containing the FCx was sliced in the coronal plane (300 µm) with a vibratome (Leica, Nussloch, Germany) in ice-cold low Ca^2+^ artificial cerebrospinal fluid (ACSF) containing (in mM): 126 NaCl, 2.5 KCl, 2 CaCl_2_, 2 MgCl_2_, 1.25 NaH_2_PO_4_, 26 NaHCO_3_, and 10 D-glucose (pH 7.3–7.4). Slices (two per animal) were transferred in a holding chamber and allowed to recover for at least 1 hr before being placed in the recording chamber and superfused with ACSF (32–34°C) saturated with 95%O_2_/5%CO_2_:

FCx layer II/III pyramidal cells were identified visually with an upright microscope with infrared illumination, and whole-cell voltage-clamp recordings were made by using an Axopatch 200B amplifier (Molecular Devices, CA). All GABA_A_ IPSC recordings were made with electrodes filled with an internal solution containing the following (mM): 140 cesium-methylsulfonate, 0.2 EGTA, 5 NaCl, 10 HEPES, 2 Mg_2_ATP, 0.25 Mg_2_GTP, pH 7.2–7.4. Experiments were begun only after series resistance had stabilized (typically 15–40 MΩ). Series resistance and input resistance were monitored continuously on-line with a 4 mV depolarizing step (25 ms). Data were filtered at 2 KHz, digitized at 10 KHz, and collected on-line with acquisition software (Clampex 8.2, Molecular Devices, CA). Neurons were voltage-clamped at a membrane potential of 0 mV. All GABA_A_ spontaneous IPSCs were recorded in presence of 2-amino-5-phosphonopentanoic acid (AP5; 100 µM), 6-cyano-2,3-dihydroxy-7-nitro-quinoxaline (CNQX; 10 µM), to block N-methyl-D-aspartate- (NMDA), α-amino-3-hydroxy-5-methyl-isoxazolepropionic acid- (AMPA) mediated synaptic currents, respectively. As already described [12], there was no effect of this solution on the holding current of the pyramidal cells.

### N-acylethanolamine Quantification

Male C57BL/6J mice (Harlan, San Pietro al Natisone, Italy) were fed, as described above, with a standard diet or a 0.2% fenofibrate diet. After 14 days, mice were killed and brain rapidly removed. Frontal cortex slices were obtained and immediately frozen. Frozen slices were homogenized and extracted with chloroform/methanol/Tris-HCl 50 mM pH 7.5 (2∶1:1, v/v) containing internal deuterated standards for PEA and OEA quantification by isotope dilution ([2H]4 PEA, [2H]4 OEA; Cayman Chemicals, MI, USA). The lipid-containing organic phase was dried down, weighed and pre-purified by open bed chromatography on silica gel. Fractions were obtained by eluting the column with 90∶10 (v/v) chloroform/methanol. PEA and OEA were quantified by liquid chromatography-atmospheric pressure chemical ionization-mass spectrometry (LC-APCI-MS) [(Agilent 1100 HPLC system (Agilent, Palo Alto, CA, USA) equipped with MS Detector 6110 single quadruple)], and using selected ion monitoring at M +1 values for the four compounds and their deuterated homologues, as previously described [26], [27].

### Phosphorylation of nAChRs

Phosphorylation of the β2*-nAChRs by the PPARα agonist was assessed *ex vivo* by immunoblotting in rat brain homogenates. Rats (250–300 g) were treated with the PPARα agonist WY14643 (40 mg/kg, i.p.) or vehicle and killed after 15 min. Brains were rapidly removed and the FCx was immediately frozen in liquid nitrogen. The tissue was then sonicated in cell lysis buffer (50 mM TRIS, pH 7.4, 250 mM NaCl, 5 mM EDTA, 50 mM NaF, 1 mM sodium orthovanadate, 1% Triton X-100, 0.02% NaN_3_) containing1 mM phenylmethylsulfonyl fluoride and protease inhibitor cocktail. Protein concentrations of the lysates were measured by the Bio-Rad Dc Protein Assay.

β_2_ subunit protein was immunoprecipitated from whole-cell lysates using a rabbit polyclonal antibody raised against a recombinant protein corresponding to amino acids 342–433 of the human β_2_ subunit (sc-11372, Santa Cruz Biotechnology). Antibodies were coupled to protein A Dynabeads (Invitrogen) using 5 µg anti-β_2_ antibody by rotating the mixture for 10 min at room temperature. Beads were washed twice in PBS and then the antibody-conjugated beads were incubated with 500 µg of protein lysate for 10 min at 4°C, followed by 3 washing steps in PBS supplemented with 0.1% Tween 20 (Sigma-Aldrich). Bound protein was eluted with 4× XT Sample buffer (Bio-Rad) and Reducing agent (Bio-Rad).

The immuno-precipitates were separated on an XT Criterion 10% gel (Bio-Rad, Copenhagen, Denmark) with 1× XT MOPS running buffer (Bio-Rad) for 1 hour at 175 V (constant) and subsequently electro-transferred to a nitrocellulose membrane at 400 mA (constant) for 1 hour. The membranes were incubated with 4G10 anti-phosphotyrosine (PY) antibodies (Upstate Biotechnology).

Chemiluminescence was detected and quantified with the Versa Doc 1000 Imaging System (Bio- Rad Laboratories, Hercules, CA, USA). Samples from control and treated rats were run on the same immunoblots and then analyzed together. Values obtained from treated rats were calculated as percentage of control values.

### Drugs

Nicotine [(−)-nicotine hydrogen tartrate)] and fenofibrate were purchased from Sigma (Italy). Nicotine was dissolved in 0.9% NaCl solution and the pH was adjusted to 7.0 with 0.1 M NaOH. Concentrations were adjusted for the mouse to receive 10 µl/g of nicotine solution. WY14643 and MK886 were purchased from Tocris. WY14643 and MK886 were dissolved in a solution containing 10% Tween80, 20% dimethyl sulfoxide (DMSO) and 70% distilled water. All drugs for patch-clamp experiments were dissolved in DMSO as stock solutions and then diluted to the final volume in ACSF (final concentration <0.01%).

### Statistical Analysis

Seizure scores (expressed as mean ±95% confidence interval, C.I.) were analyzed by utilizing Kruskal-Wallis test for non-parametric data, and Dunn’s test as a post-hoc.

Electrophysiological experiments were sampled on line and off line with data analysis electrophysiology software by computers connected to specific interface. Drug-induced changes in sIPSCs were calculated by averaging the effects following drug administration and normalized to the pre-drug baseline.

Data were analyzed utilizing parametric one-way ANOVA or Student’s t-test, when they had equal variance and were normally distributed; nonparametric t-test was utilized in all other circumstances. *Post hoc* multiple comparisons were made using the Dunnett’s or Newman-Keuls’test when appropriate. Western blot data were analyzed using Student’s t-test. Contingency tables were analyzed using Fisher’s exact test. Alpha was set at P<0.05. All analyses were performed using the software Statistica 6 (StatSoft inc. Tulsa, OK, USA).

## Results

### Measurement of Nicotine-induced Seizures

Nicotine was subcutaneously administered at the doses of 5 (n = 4), 7 (n = 5), and 10 mg/kg (n = 18) in mice and its effects were observed during 5 min after injection by two observers blind to the treatment. Data for behavioral observations were also obtained from mice carrying electrodes for EEG recordings, and were pooled with those obtained from non-implanted animals.

**Figure 1:**
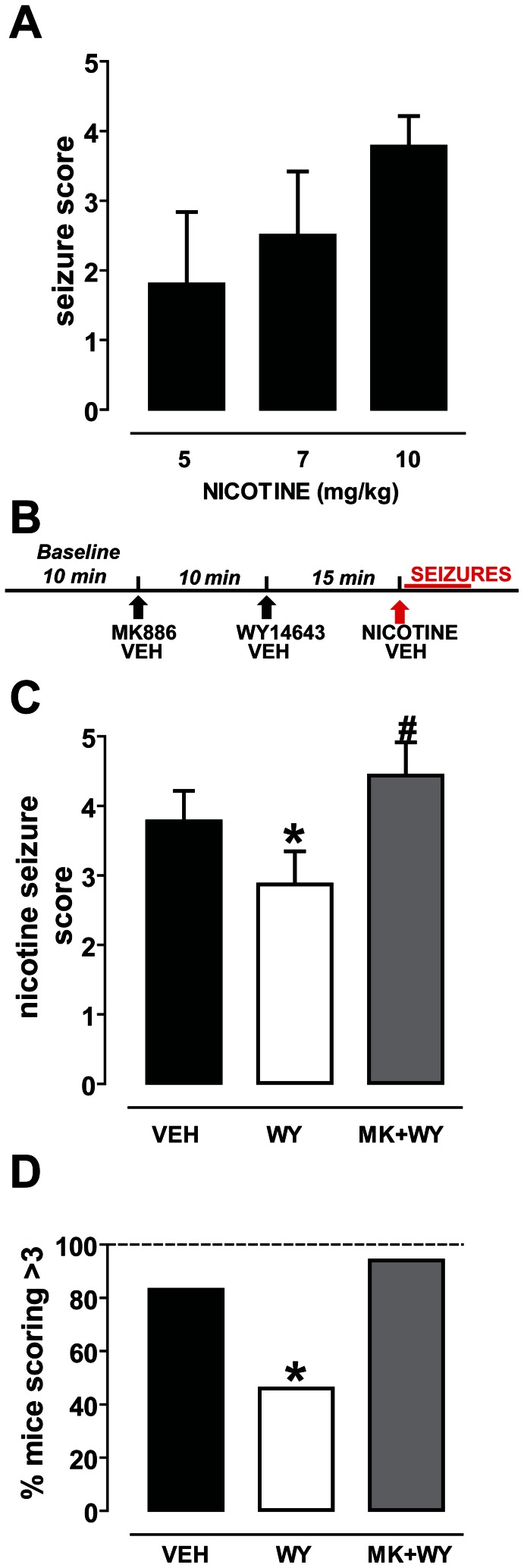
The PPARα agonist WY14643 reduces nicotine-induced seizures. (**A**) Graph displaying the dose-dependency of the severity of nicotine-induced seizures in C57BL/6 mice. Nicotine was administered subcutaneously at 5, 7 and 10 mg/kg (n = 4, n = 5, n = 18, respectively). (**B**) Schematic representation of the experimental protocol of experiments in (C) and (D). (**C**) The PPARα agonist WY14643 (WY, 80 mg/kg, i.p., n = 23) reduced the severity of nicotine-induced seizures (nicotine dose: 10 mg/kg, s.c) (*P<0.05 vs. vehicle, Dunn’s-test). This effect was abolished by the selective PPARα antagonist MK886 (MK, 3 mg/kg, i.p.). (n = 16, # P<0.001 vs. WY, Dunn’s-test). (**D**) This graph shows that the percentage of mice undergoing severe nicotine-induced seizures (indexed by scores >3) is significantly attenuated after WY14643 pretreatment (*P<0.05 vs. vehicle, Fisher’s test). MK886 reversed this effect (P>0.05 vs. vehicle, Fisher’s test). Data are expressed as mean±95% C.I.

After a first exploratory phase, mice displayed impaired locomotor activity with shakes. A few seconds later they developed strong tachypnea with tremors, back arching, and partial loss of righting reflex. At higher doses, the previous symptoms were followed by increased locomotor activity, with rapid movements of the legs and wild running. Clonic and tonic seizures occurred after complete loss of righting reflex. Fig. 1A shows that nicotine elicited seizures with dose-dependent severity (r^2^ = 0.76, P<0.001). The calculated ED_50_ for nicotine was 5.08±0.07 mg/kg, similar to that already reported in the literature for the same mouse strain [24]. Only the dose of 10 mg/kg was able to induce severe symptoms, which scored higher than 3. In this case, the number of animals which scored 4 or 5 was 15 out of 18 (83%) and, therefore, we chose this dose for the subsequent experiments.

### The PPARα agonist WY14643 Acutely Reduces the Severity of Nicotine-induced Seizures

To test the effect of the PPARα agonist WY14643 (WY, 80 mg/kg, i.p.) and the antagonist MK886 (MK, 3 mg/kg, i.p.) on nicotine-induced seizures, mice were randomly assigned to three groups: i) the vehicle-vehicle-nicotine (VEH group), ii) the vehicle-WY-nicotine group (WY group), and iii) the MK886-WY-nicotine group (MK-WY group). Each animal received the three injections spaced 10 and 15 min (Fig. 1B).

When scores were evaluated, non-parametric ANOVA yielded a highly significant difference among groups (P<0.0001, Kruskal-Wallis test) (Fig. 1C). Post-hoc analysis revealed that mice pre-treated with the PPARα agonist WY14643 were significantly protected against nicotine-induced seizures when compared with vehicle pre-treated animals. The difference in the average score was statistically significant (VEH mice: 3.8±0.4, n = 18; WY mice: 2.9±0.5, n = 24; P = 0.019, Dunn’s test) (Fig. 1C). Additionally, WY mice which obtained a score of 4 or 5 were 46% of all treated mice (11 out of 24). The difference was statistically significant when compared with the VEH-group (15/18, 83%) (P<0.05, Fisher’s test, Fig. 1D). The protective effect of WY was reverted by the PPARα antagonist MK. Hence, post-hoc analysis indicated that in the MK-WY-group the severity of nicotine-induced seizures was restored, since the average score was significantly higher than that assigned to the WY mice (4.4±0.5, n = 16, P = 0.001, Dunn’s test, Fig. 1C), but not different from that of VEH-mice (P>0.05, Dunn’s test) (Fig. 1C). Consistently, MK-WY mice receiving a score of 4 or 5 were 15 out of 16 (94%) (P = 0.6 vs. VEH mice) (Fig. 1D).

*In vivo* electrophysiological recordings revealed that high doses of nicotine elicited seizures that originate in the hippocampus [28] and in the thalamo-cortical pathways [11]. These neural circuits may also be involved in NFLE (for review, see [29]). For this reason, one EEG electrode was placed in the sensorimotor cortex and two in the dorsal hippocampus. Of the two electrodes positioned in the hippocampus the one with the best signal was chosen.

**Figure 2:**
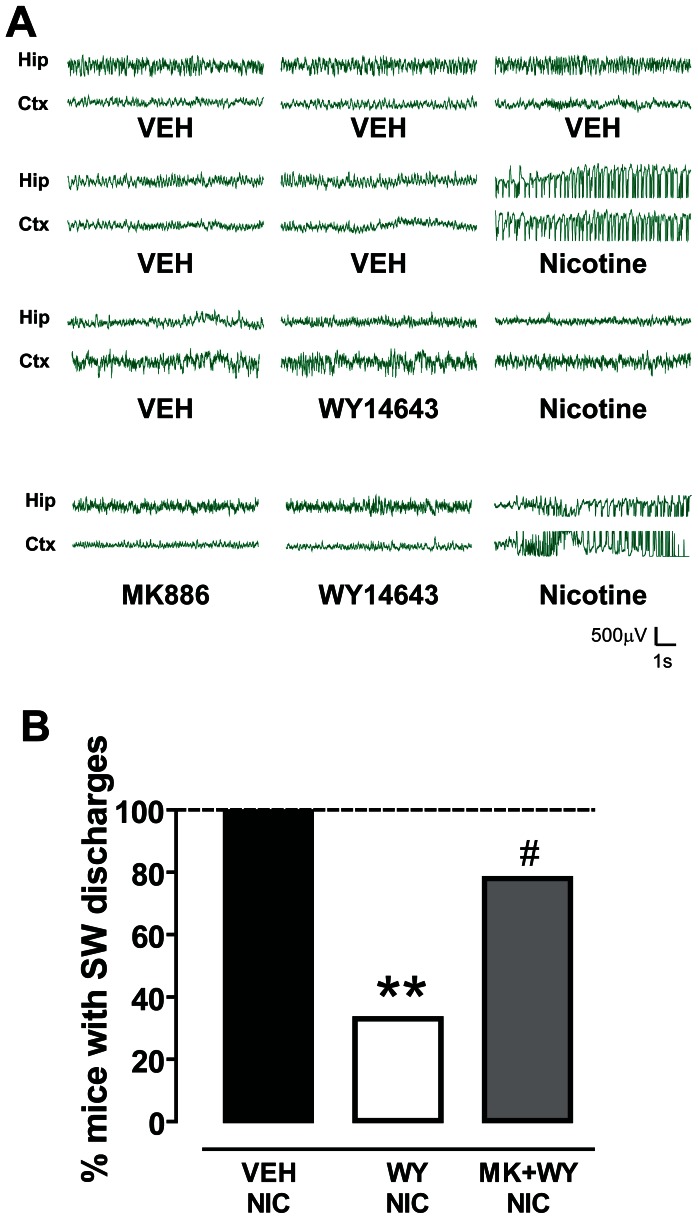
The PPARα agonist WY14643 suppresses nicotine-induced spike-wave activity. (**A**) Representative traces of EEG recordings from hippocampal (Hip) and sensorimotor cortical (Ctx) electrodes chronically implanted in mice. Following the administration of vehicle (VEH) and 10 mg/kg nicotine, bursts of synchronous spike-wave (SW) activity with high-amplitude and low-frequency (most in the delta rhythm range) were recorded. This activity was suppressed when animals were pretreated with the PPARα agonist WY14643 (WY, 80 mg/kg), which *per se* did not change baseline EEG activity. The PPARα antagonist MK886 (MK, 3 mg/kg, i.p.) restored nicotine-induced SW discharges. (**B**) The graph shows the percentage of mice presenting SW discharges following the three treatment protocols. Vehicles treated mice did show SW activity, whereas 100% of nicotine treated mice displayed bursts of SW activity. The effects of nicotine were blocked in the majority of WY treated mice, since SW burst were recorded only in 33% of treated animals (**P<0.01 vs. vehicle, Fisher’s test). Conversely, when MK was administered 15 before WY nicotine-induced SW activity was recorded in 78% of MK+WY treated mice (^#^P<0.05 vs. WY, Fisher’s test).

As depicted in Fig. 2, nicotine-induced (10 mg/kg, s.c.) convulsive activity was paralleled by a synchronous spiking discharge pattern both in the hippocampus and in the cortex in 7 out of 7 (100%) treated mice (Fig. 2A, B). Spiking activity had an onset of 130±7 s after nicotine injection and a duration of 52±10 s (n = 7). No spike/wave discharge events were recorded in vehicle treated mice (Fig. 2 A, B).

Pretreatment with WY significantly attenuated the effects of nicotine, since only 3 out of 9 (33.3%) mice displayed spiking activity when compared with vehicle-treated mice (P<0.05, Fisher’s test, Fig. 2B). WY pretreatment, however, did not change either the onset or the duration of spike-wave activity in those animals not responding to the treatment (114±66 s and 32±18 s, respectively, n = 3). Consistent with behavioral experiments, MK prevented WY-induced protection from seizures, since nicotine’s effect was restored in 7 out of 9 mice (77.8%) (Fig. 2A, B).

### Chronic Administration of the Clinically Available PPARα agonist Fenofibrate Attenuates the Severity of Nicotine-induced Seizures

The results of the previous experiments prompted us to assess the efficacy of the clinically available PPARα agonist fenofibrate. Fenofibrate does not cross the blood-brain barrier rapidly enough to allow acute studies *in vivo*; therefore, we chronically treated the animals with a diet containing 0.2% w/w fenofibrate [30]. Mice were randomly divided into three groups: (i) fenofibrate diet (FBR), (ii) control diet (CTRL) and (iii) fenofibrate washout diet (FBR-WO). The fenofibrate containing diet was administered ad libitum for 14 days. FBR mice consumed a daily average of 3.6±0.1 g of food pellets, which approximately corresponds to 28–30 mg/kg fenofibrate per day. FBR mice were divided in two additional groups at the end of the treatment: one group received the PPARα antagonist MK886 15 min before the nicotine challenge, and the other received its vehicle. CTRL mice were fed with the control diet, identical and equicaloric but without fenofibrate. FBR-WO mice were fed for 14 days with a fenofibrate-containing diet, as FBR mice, and then fed with a control diet for additional 14 days to assess the effect of fenofibrate washout. In each animal EEG electrodes were implanted 7 days before the beginning of the treatments and nicotine was tested on the 15^th^ day from the beginning of treatment (for FBR and CTRL mice) or on the 30^th^ day (for FBR-WO mice).

**Figure 3:**
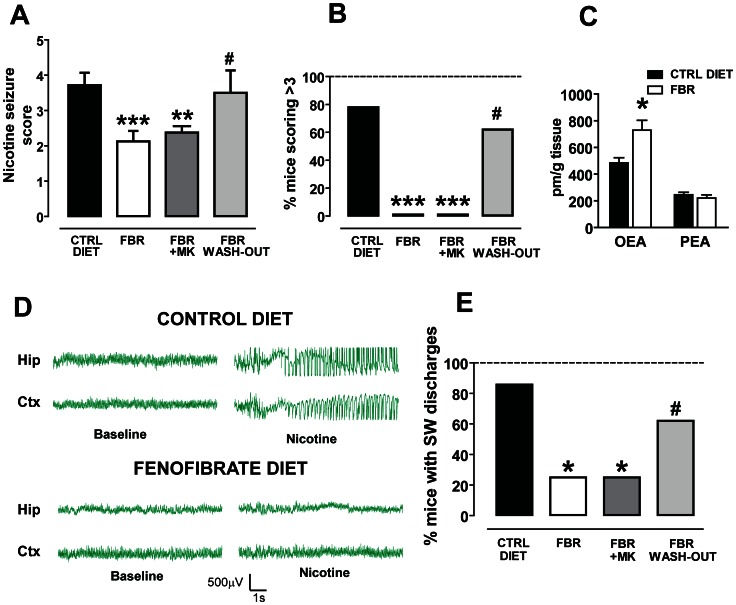
The clinically used PPARα agonist fenofibrate chronically administered with food reduces nicotine-induced seizures and spike-wave activity, and increases the OEA levels in the frontal cortex. (**A**) Graph displaying that fenofibrate (n = 8, FBR, 0.3% w/w in the diet for 14 days) reduced the severity of nicotine-induced seizures (nicotine dose: 10 mg/kg, s.c) (***P<0.001 vs. control diet, CTRL DIET, Dunn’s-test). This effect was not abolished by the selective PPARα antagonist MK886 (FBR+MK, 3 mg/kg, i.p.) (n = 8, **P<0.01 vs. CTRL DIET, Dunn’s-test) but by withdrawal of fenofibrate treatment for 14 days (FBR WASH-OUT, n = 8, # P<0.05 vs. FBR and P>0.05 vs. CTRL DIET, Dunn’s test). (**B**) This graph shows that the percentage of mice undergoing severe nicotine-induced seizures (indexed by scores >3) is significantly attenuated after chronic fenofibrate treatment (***P<0.001 vs. control diet, Fisher’s test) and restored after fenofibrate withdrawal for 14 days (#P>0.05 vs. control diet). MK886 did not reverse this effect (***P<0.001 vs. control diet, Fisher’s test). (**C**) Chronic activation of PPARα by fenofibrate changes oleoylethanolamide (OEA), but not palmitoylethanolamide, levels within frontal cortex. Concentrations of these endogenous PPARα ligands are expresses as pmol per gram of tissue. Error bars depict S.E.M. (*P<0.05, Student’s t-test). (**D**) Representative traces of EEG recordings from hippocampal (Hip) and sensorimotor cortical (Ctx) electrodes chronically implanted in mice. In mice fed with control diet 10 mg/kg nicotine elicits bursts of synchronous spike-wave (SW) activity with high-amplitude and low-frequency (most in the delta rhythm range). This activity was suppressed in animals fed with fenofibrate in food pellets. (**E**) The graph shows the percentage of mice presenting SW discharges following the four treatment protocols. 86% of control diet fed mice (6 out of 7) did show nicotine-induced SW activity. The effects of nicotine were fully blocked in all fenofibrate treated mice, since SW burst were recorded only in none of treated animals (***P<0.01 vs. control diet, Fisher’s test). MK, administered 15 before nicotine, did not restore nicotine-induced SW activity (***P<0.001 vs. control diet, Fisher’s test), whereas fenofibrate washout did (#P>0.05 vs. control diet, Fisher’s test). Data are expressed as mean±95% C.I.

When nicotine-induced seizures were scored, non-parametric ANOVA yielded a highly significant difference among groups (P<0.0001, Kruskal-Wallis test) (Fig. 3A). Post-hoc analysis revealed that FBR mice were significantly protected against nicotine-induced seizures when compared with CTRL animals. The difference in the average score was statistically significant (CTRL mice: 3.4±0.7, n = 14; FBR mice: 2.1±0.3, n = 8; P<0.001, Dunn’s test) (Fig. 3A). Additionally, none among the FBR mice obtained a score of 4 or 5 (0 out of 8) (Fig. 3 B). The difference was statistically significant when compared with the CTRL group (11/14, 78%) (P<0.001, Fisher’s test, Fig. 3B). The protective effect of the fenofibrate diet was not reverted by the PPARα antagonist MK. Hence, post-hoc analysis indicated that in the MK-treated FBR mice the severity of nicotine-induced seizures was not restored, since the average score was significantly lower than that assigned to CTRL mice (2.4±0.4, n = 8, P<0.001, Dunn’s test, Fig. 3A), but not different from that of FBR-mice (P>0.05, Dunn’s test) (Fig. 3A). Consistently, no MK-treated FBR mice received a score of 4 or 5 (Fig. 3B).

The lack of effect by MK could be explained by the fact that PPARα activation induces mitochondrial activity, peroxisomal β-oxidation and the biosynthesis of endogenous PPARα ligands, the N-acylethanolamines OEA and PEA. These combined effects might trigger a feed-forward mechanism to further sustain PPARα activity [31]. To test this hypothesis we measured the levels of OEA and PEA in the FCx from FBR and CTRL mice. As predicted, in FBR mice OEA levels were significantly increased in the FCx when compared to CTRL mice (729.5±73.4 pm/g vs. 484.3±38.4 pm/g; n = 5; P<0.05, Student’s t-test) (Fig. 3C). No changes of PEA levels were detected (FBR: 221.7±23.0 pm/g; CTRL: 245.6±18.4 pm/g; n = 5; P = 0.44 Student’s t-test) (Fig. 3C).

Fenofibrate washout for 14 days abolished the protective effects of the drug, since in FBR-WO mice the severity of nicotine-induced seizures was restored (Fig. 3A). FBR-WO mice received a score of 3.5±0.6 (n = 8), significantly higher that FBR mice (P<0.05, Dunn’s test) but not dissimilar from CTRL mice (P>0.05, Dunn’s test) (Fig. 3A).

EEG recordings revealed that nicotine-induced (10 mg/kg, s.c.) convulsive activity was paralleled by a synchronous spiking discharge pattern in both the hippocampus and the cortex in 6 out of 7 (86%) CTRL mice (Fig. 3D, E). Spiking activity had an onset of 148±8 s after nicotine injection and a duration of 53±9 s (n = 6).

The effect of nicotine was significantly attenuated in FBR mice, since only 2 out of 8 (25%) mice displayed spiking activity, when compared with CTRL mice (P<0.05, Fisher’s test, Fig. 3E). In FBR mice, however, neither the onset nor the duration of spike-wave activity changed in those animals not responding to the treatment (179.5±25 s and 27±2 s, respectively, n = 2).

The effects of nicotine were restored in FBR-WO mice: spiking activity was indeed present in 5 out of 8 mice (P>0.05 vs. CTRL mice, Fisher’s test) (Fig. 3E). Consistent with behavioral experiments, MK did not block FBR-induced protection from seizures, since nicotine’s spike-wave activity was assessed in 2 out of 8 mice (25%) (Fig. 3E).

### PPARα Agonists Suppress Nicotine-induced sIPSCs in Layer II–III Pyramidal Neurons in the Mouse and Rat Frontal Cortex

Nicotine-induced seizures, as well as those spontaneously occurring in transgenic mice carrying the NFLE mutations, might be primarily generated within FCx circuits [12], [29]. Since excitatory transmission on FCx pyramidal neurons is not affected by NFLE mutations or by nicotine [12], we carried out whole-cell recordings of spontaneous inhibitory postsynaptic currents (sIPSCs) from layer II/III pyramidal cells of mouse and rat coronal slices.

**Figure 4:**
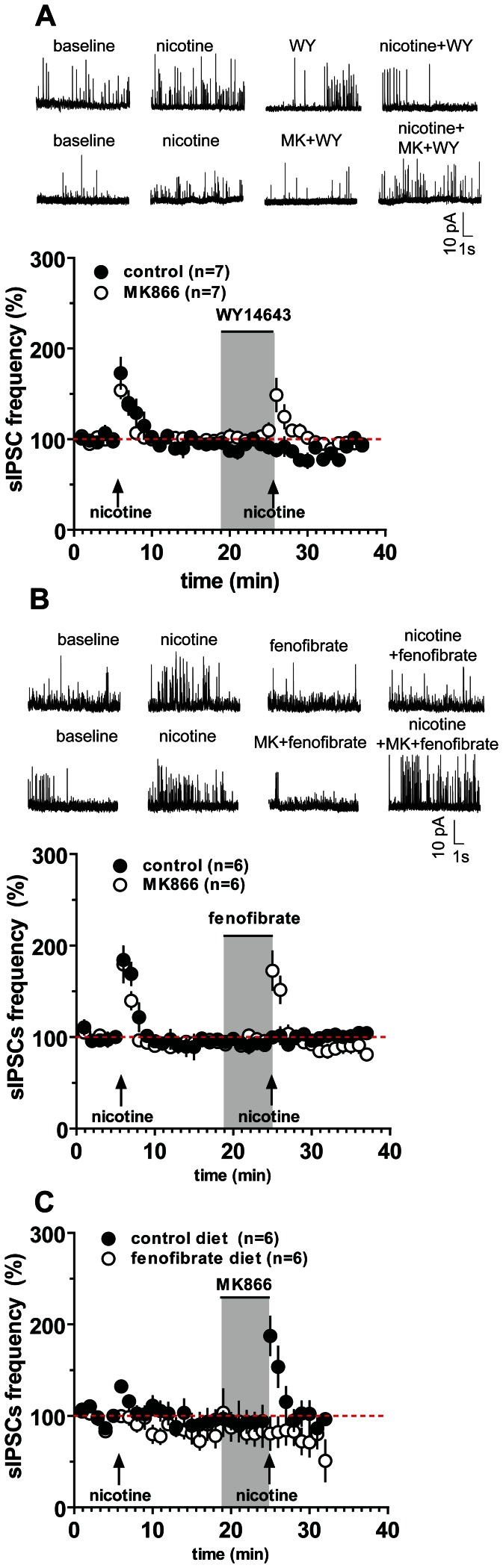
The PPARα agonists WY14643 and fenofibrate suppress nicotine-induced increase of spontaneous inhibitory postsynaptic currents (sIPSC) in frontal cortex (FCx) pyramidal neurons. The graphs illustrate that in mouse FCx slices, nicotine (5 µM perfused at arrows for 30 s) increases sIPSCs frequency in layer II/III pyramidal neurons. Acutely, the PPARα agonists WY14643 (1 µM, WY) (**A**), and fenofibrate (10 µM) (**B**) fully suppressed nicotine-induced increase in sIPSC frequency. Similarly, chronic fenofibrate (0.2% w/w for 14 days in food) fully suppressed nicotine-induced increase in sIPSC frequency (**C**). The gray box represents the time of PPARα agonist (+/− antagonist) perfusion. The PPARα antagonist MK886 (0.3 µM) (open symbols) blocked the effects of acute WY (**A**) and fenofibrate (**B**), but not the one of chronic fenofibrate (**C**), and restored nicotine-induced increase in sIPSCs. Representative recordings of the effect of nicotine, and of PPARα agonists and antagonist, on spontaneous IPSCs from pyramidal cells at V_h_ = 0 mV are depicted on the upper part of panels A and B. Symbols represent the average±S.E.M.

Under voltage-clamp mode (V_holding_ = 0 mV to isolate sIPSCs [12]), nicotine (5 µM, 30 s) significantly increased both frequency and amplitude of sIPSCs in mouse pyramidal cells. sISPC frequency was enhanced to 133.2±2.9% of baseline (P<0.0001, n = 23, paired t-test), the amplitude to 116.5±7.5% of baseline (P<0.05, n = 23, paired t-test) (Fig. 4, Table 1). In marked contrast, pretreatment with the PPARα agonists WY (1 µM, 5 min) and fenofibrate (10 µM, 5 min) dramatically blunted the effect of nicotine on sIPSCs. During nicotine application in the presence of WY, sIPSC frequency was 91.8±10.2% of baseline and amplitude was 91.8±4.7% of control values (for both parameters, P>0.05 vs. baseline, n = 7, paired t-test) (Fig. 4A, Table 1). Consistently, the structurally different PPARα agonist fenofibrate also suppressed nicotine-induced increase of sIPSC frequency and amplitude (for both parameters, P>0.05 vs. baseline, n = 6, paired t-test) (Fig. 4B, Table 1).

**Table 1:** Table showing frequency and amplitude of spontaneous IPSCs recorded from mice and rat pyramidal neurons in layers II/III of the frontal cortex.

	Baseline	Nicotine	WY +Nicotine	FBR +Nicotine	WY+MK+Nicotine	FBR+MK+Nicotine
**C57BL/6J mice**						
Frequency, Hz	46.2±1.0	61.2±1.1***	35.1±6.6	40.0±1.6	68.7±12.2*	79.7±16.7*
Amplitude, pA	17.8±0.8	19.8±0.8*	18.3±2.7	18.1±1.6	21.5±1.9*	20.3±1.5*
**Rats**						
Frequency, Hz	15.3±3.2	22.2±4.7***	15.8±11	11.5±0.6	22.5±5.7**	22.5±7.7*
Amplitude, pA	16.4±0.7	21.3±1.5***	13.4±0.8	14.0±1.3	25.1±5.4*	22.8±1.9*

Nicotine (5 µM) significantly increases both frequency and amplitude of sIPSC (*P<0.05, 01, ***P<0.001 vs baseline, paired t-test). Both WY14643 (WY 1 µM) and fenofibrate (FBR, 10 µM) suppressed nicotine’s effects on sIPSCs frequency and amplitude. The PPARα antagonist MK886 restored nicotine’s effects on sIPSC frequency (*P<0.05, **P<0.01 vs baseline, paired t-test).

The effects of both PPARα agonists were blocked by the synthetic PPARα antagonist MK. In the presence of MK (0.3 µM, 5 min) and either PPARα agonist, nicotine effects on sIPSCs frequency and amplitude were fully restored. Hence, in slices perfused with WY+MK or fenofibrate+MK, nicotine induced an increase in sIPSC frequency to 141.7±19.3% and to 172.5±20.9% of baseline, respectively (P>0.05vs. nicotine alone, n = 6–7, paired t-test; Table 1, Fig. 4A,B). Table 1 shows that sIPSC amplitude in the presence of nicotine alone was not significantly different from the amplitude in the presence of nicotine+WY+MK or nicotine+fenofibrate+MK(P>0.05, n = 6, paired t-test).

To assess whether suppression of nicotine effects by acute PPARα agonists was species-specific we replicated the experiments described above in Sprague Dawley rats. The results (Supporting Results S1) confirmed that both WY and fenofibrate abolished nicotine-induced effects in both species (Table 1; Figure S1).

Experiments with mice chronically fed with fenofibrate (0.2% w/w for 14 days) confirmed that this drug abolishes nicotine-induced increases in sIPSC frequency in FCx pyramidal neurons. Hence, nicotine-induced increase in sIPCS frequency was 132.2±4.6% of baseline (n = 6) in CTRL mice vs. 99.7±5.6% of baseline (n = 6) in FBR mice (P<0.05, Student’s t-test) (Fig. 4C). Consistent with behavioral experiments, MK was unable to revert the effect of fenofibrate, since in FBR mice MK did not restore the effects of nicotine (sIPSC frequency in control animals was 187.5±20.9% vs. 80.7±17.6% in FBR mice).

### The PPARα Agonist Enhances Phosphorylation of the β2 Subunit of nAChRs

**Figure 5:**
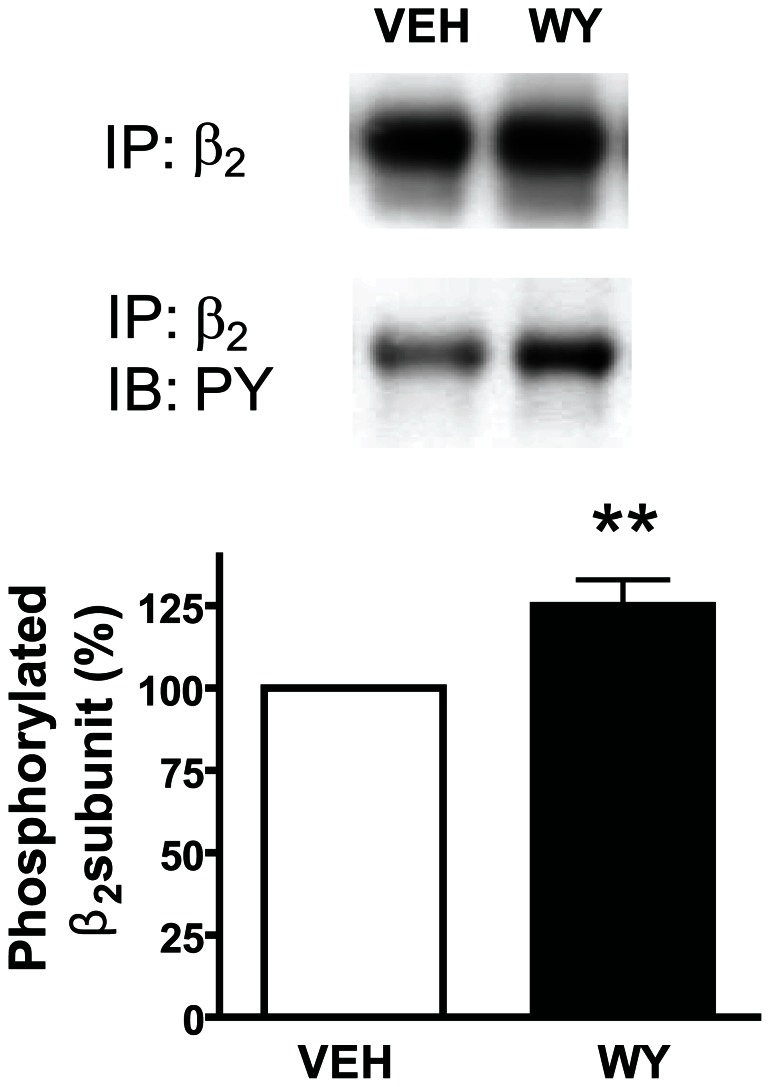
The PPARα agonist WY14643 enhances phosphorylation of β2 subunits of the nicotinic acetylcholine receptors. Graph and representative immunoblots showing that the PPARα agonist WY (40 mg/kg, i.p.) caused an increase in β2-subunit phosphorylation (*P<0.05) in rat frontal cortex homogenates. Tissue lysates were subjected to immunoprecipitation (IP) with anti-β_2_ subunit antibody and were immunoblotted (IB) with anti-phosphotyrosine (PY) antibody. The blots were stripped and reprobed with anti-β_2_ subunit antibody to normalize for protein loading.

Consistent with our previous reports [13], [14], these findings led us to hypothesize that PPARα might regulate the balance between phosphorylated and dephosphorylated β2*nAChRs. To test this hypothesis, we analyzed phosphorylation of β2 subunits with Western blot after *in vivo* exposure to the synthetic PPARα agonist WY. Rats were administered WY (40 mg/kg, i.p.) and after 15 min, brain were rapidly removed and the FCx were dissected. As expected, in FCx total homogenates an increased immunoreactivity for phosphorylated β2 subunits was observed in WY-treated animals (125.0±7.9% of controls, n = 7, p<0.01, Student’s t-test) (Fig. 5).

## Discussion

Our study shows that acute and chronic PPARα agonists, including the clinically available fenofibrate, reduce nicotine-induced behavioral and EEG seizure expression and abolish nicotine-induced enhancement of sIPSCs in FCx pyramidal neurons.

We presume, based on the present results and previous extensive work by our group, that the mechanism whereby PPARα exerts these effects involves phosphorylation of specific nAChR subunits [13], [14], [22], [23]. Indeed, nAChR channel properties depend on its tyrosine phosphorylation status, being the result of a balance between tyrosine kinases and phosphatases [32], which negatively or positively modulates nAChR-mediated currents, respectively, and controls the number of functional surface receptors [33].

We specifically measured the ratio of phosphorylated/dephosphorylated β2 subunits, which is increased in the FCx by acute PPARα ligand treatment, but it is difficult to quantify this effect in subunit combinations less abundantly expressed in the CNS. Therefore, we cannot exclude that subunits other than the β2 might also be targeted by PPARα. However, it must be pointed out that β2*nAChRs play a major role, yet not exclusive, in nicotine-induced seizures [10], [34]. In fact, the α4β2 subunit combination is the most abundantly expressed in the whole brain and particularly in regions relevant to ictogenesis, such as thalamo-cortical circuits and FCx (see [12] and references therein). Furthermore, it was shown by Klaassen et al. [12] that nicotine-induced sIPSC in layer II/III of the FCx are mediated by α4β2 nAChRs, as they are insensitive to the α7-selective blocker methyllycaconitine but fully blocked by the α4β2-selective antagonist dihydro-β-erythroidine. However, it must be pointed out that other nAChR subunits are implicated in nicotine-evoked seizures. In particular, their severity is reduced in mice lacking α3, α5, and β4 [35], [36], but not α7 [24]. The role of α7, however, is still controversial, since pharmacological experiments with selective α7 agonists and antagonists yielded contradictory results [10], [34].

Irrespectively of the subunits involved other than β2, the PPARα agonists reduced both the severity of nicotine-induced effects and the number of animals undergoing severe symptoms. These behavioral effects were paralleled by a significant decrease in the number of mice that showed bursts of spike-wave discharges. The pharmacological specificity was confirmed by the finding that pre-treatment with the PPARα antagonist prevented the protection exerted by the agonist, with the exception of chronic fenofibrate treatments. The latter finding might be explained with the finding that PPARα activation induces fatty acid metabolism [31] and NAE biosynthesis with increased production of OEA. Notably, OEA would further sustain PPARα activation by triggering a feed-forward mechanism difficult to be antagonized by a single administration of the PPARα antagonist MK. A second possible explanation is that changes induced by this treatment regimen, possibly on function, number and phosphorylation status of nAChRs, are not promptly reversed by an acute treatment with the PPARα antagonist. Indeed, a reversal of fenofibrate-induced protection is observed following a 14-days washout of the drug, achieved by replacing fenofibrate-containing food pellets with control food.

The present data, while confirming the relationship between nAChRs and epileptogenesis, give support to the role played by nuclear receptors PPARα in the modulation of nAChR function in the CNS. Hence, in our previous studies, we demonstrated that both endogenous and synthetic PPARα agonists suppressed nicotine-induced electrophysiological effects on dopamine neurons and prevented behavioral effects of nicotine predictive of its addicting properties by regulating β2* nAChRs specifically [13], [14], [15], [16]. Here we extend those results to the seizure-inducing action of nicotine.

Nuclear and cytoplasmic immunostaining for PPARα is present in neurons distributed in all layers of the FCx [20], [21], whereas cholinergic afferents, arising principally from the basal forebrain, innervate neurons in all layers of the rodent FCx [37]. Several subtypes of GABA cortical interneurons express functional α4β2* nAChRs [38], [39] and have been involved in nicotine-induced seizures [10], [12], [34], [38] since they innervate adjacent pyramidal cells. Consistently, we show that nicotine induced an increase in the frequency of GABA_A_-mediated sIPSCs, possibly by depolarizing presynaptic GABAergic terminals impinging on pyramidal neurons. According to behavioral observations, these effects were suppressed by PPARα agonists, suggesting that PPARα regulate the functions of nAChRs in cortical interneurons, similarly to dopamine neurons.

Notably, the relevance of our results might extend beyond nicotine-induced seizures or NFLE, since chronic fenofibrate was effective in rats as anticonvulsant in pentylentetrazole-induced seizures and on latencies to the onset of status epilepticus induced by lithium–pilocarpine [30]. Additionally, recent studies demonstrated that the antiepileptic drugs carbamazepine and lamotrigine, which are employed effectively also in NFLE patients, negatively modulate the activity of α4β2 nAChRs [40], [41]. This effect might contribute to their mechanisms of action since these receptors control neuronal excitability and both glutamate and GABA release in the hippocampus and the thalamo-cortical system [42], [43], [44], [45], [46].

In conclusion, we provided evidence that PPARα within the CNS might be a key regulator of neuronal activity by the modulation of functional properties of nAChRs. These effects might be therapeutically exploited not only when nicotine addiction is concerned [15], but also for idiopathic or genetically determined forms of epilepsy where nAChRs play a major role.

## Supporting Information

Figure S1 The PPARα agonists WY14643 and fenofibrate suppress nicotine-induced increase of spontaneous inhibitory postsynaptic currents (sIPSC) in rat frontal cortex (FCx) pyramidal neurons. The graphs illustrate that in rat FCx slices, nicotine (5 µM perfused at arrows for 30 s) increases sIPSCs frequency in layer II/III pyramidal neurons. The PPARα agonists WY14643 (1 µM, WY) (**A**) and fenofibrate (10 µM) (**B**) (n = 6–7; closed symbols) fully suppressed nicotine-induced increase in sIPSC frequency. The gray box represents the time of PPARα agonist (+/− antagonist) perfusion. The PPARα antagonist MK886 (0.3 µM) (open symbols) blocked the effects of WY (**A**) and fenofibrate (**B**) (n = 6) and restored nicotine-induced increase in sIPSCs. Symbols represent the mean±SEM.
(TIF)

Supporting Results S1 PPAR-α agonists suppress nicotine-induced sIPSCs in layer II-III pyramidal neurons in the rat frontal cortex.
(DOCX)
