# Supplementary figures and images for: PPAR-Alpha Agonists as Novel Antiepileptic Drugs: Preclinical Findings
Source: PLoS One. 2013 May 27;8(5):e64541. doi: 10.1371/journal.pone.0064541 (PMC3664607; doi:10.1371/journal.pone.0064541)

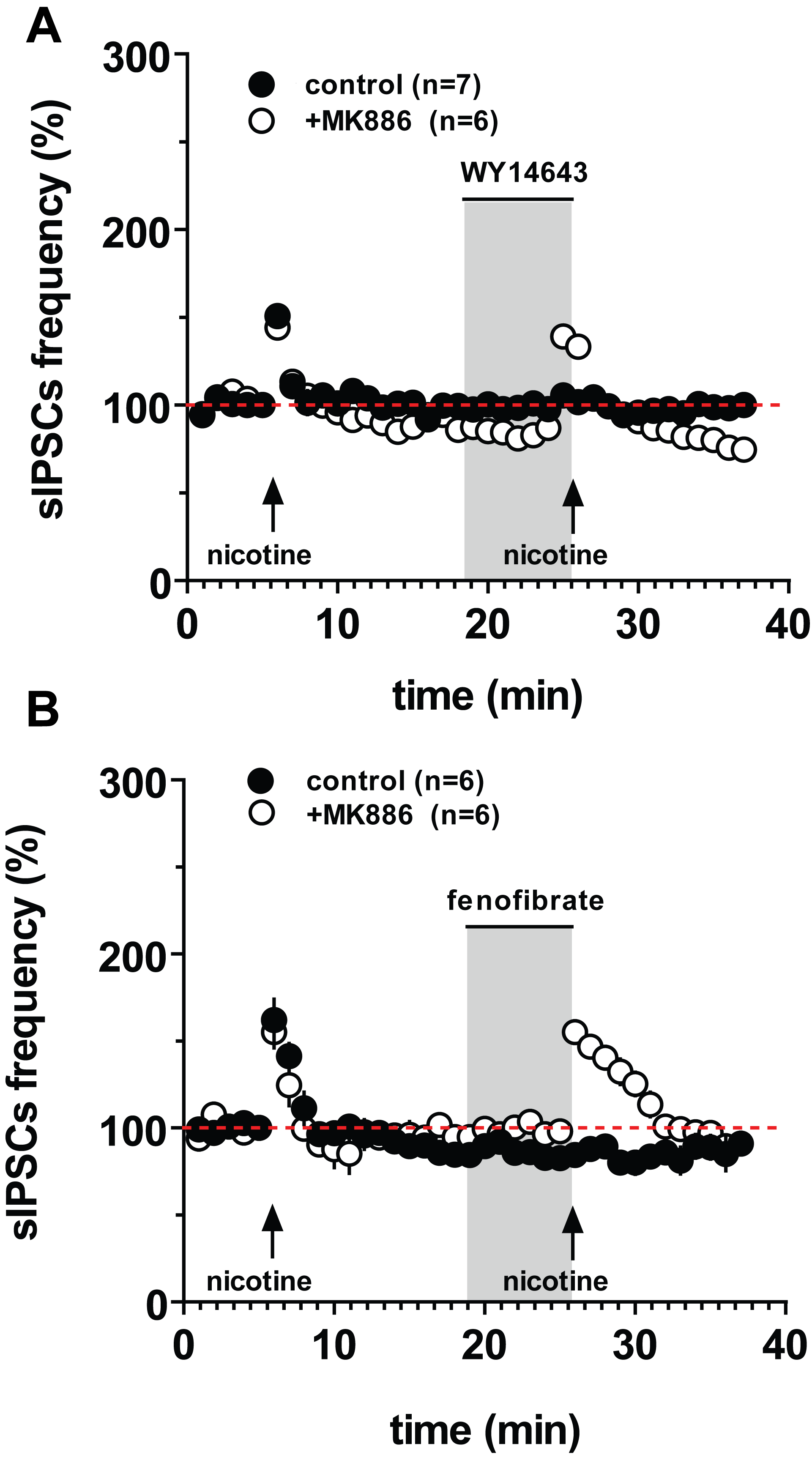

Supplement: Figure S1 — The PPARα agonists WY14643 and fenofibrate suppress nicotine-induced increase of spontaneous inhibitory postsynaptic currents (sIPSC) in rat frontal cortex (FCx) pyramidal neurons. The graphs illustrate that in rat FCx slices, nicotine (5 µM perfused at arrows for 30 s) increases sIPSCs frequency in layer II/III pyramidal neurons. The PPARα agonists WY14643 (1 µM, WY) (A) and fenofibrate (10 µM) (B) (n = 6–7; closed symbols) fully suppressed nicotine-induced increase in sIPSC frequency. The gray box represents the time of PPARα agonist (+/− antagonist) perfusion. The PPARα antagonist MK886 (0.3 µM) (open symbols) blocked the effects of WY (A) and fenofibrate (B) (n = 6) and restored nicotine-induced increase in sIPSCs. Symbols represent the mean±SEM. (TIF) [file pone.0064541.s001.tif]
