# Supplementary material for: PPAR-Alpha Agonists as Novel Antiepileptic Drugs: Preclinical Findings
Source: PLoS One. 2013 May 27;8(5):e64541. doi: 10.1371/journal.pone.0064541 (PMC3664607; doi:10.1371/journal.pone.0064541)
Supplement: Supporting Results S1 — PPAR-α agonists suppress nicotine-induced sIPSCs in layer II-III pyramidal neurons in the rat frontal cortex. (DOCX) [file pone.0064541.s002.docx]

**SUPPORTING RESULTS**

***PPAR-α agonists suppress nicotine-induced sIPSCs in layer II-III pyramidal neurons in the rat frontal cortex.***

In rats, nicotine increased both frequency and amplitude of sIPSCs in frontal cortex pyramidal cells. sISPCs frequency was enhanced to 145.1±4.5 % of baseline (P<0.0001, n=23, paired t-test, Table 1, Supplementary Fig. 1), the amplitude increased to 135.7±12.3% of baseline (P<0.05, n=23, paired t-test, Table 1). Conversely, pretreatment with the PPAR-α agonists WY (1 μM, 5 min) and fenofibrate (10 μM, 5 min) suppressed nicotine effects on sIPSCs. During nicotine perfusion in the presence of WY, sIPSC frequency was 106.3±2.9 % of baseline (P>0.05 vs baseline, n=7, paired t-test, Table 1, Supplementary Fig. 1), amplitude was 98.9±1.1% of control values (P>0.05 vs baseline, n=7, paired t-test, Table 1). Also in rat frontal cortex pyramidal neurons, fenofibrate suppressed nicotine-induced increase of sIPSC frequency and amplitude: sIPSC frequency was 90.9±5.1 of baseline (P>0.05 vs baseline, n=6, paired t-test, Table 1, Supplementary Fig. 1), amplitude was 101.9±6.5 of baseline (P>0.05 vs baseline, n=6, paired t-test, Table 1). Also in rats MK blocked the effects of both PPAR-α agonists. When MK (0.3 μM, 5 min) and either PPAR-α agonist were perfused, nicotine effects on sIPSCs frequency and amplitude were fully restored. Hence, nicotine induced an increase in sIPSC frequency to 172.3 ± 28.8 % of baseline in WY+MK experiments (P>0.05 vs nicotine alone, n=7, paired t-test, Table 1, Supplementary Fig. 1). Consistently, when fenofibrate was perfused with MK, nicotine enhanced sIPSC frequency to 146.8 ± 4.1 % of baseline (P>0.05vs nicotine alone, n=6, paired t-test; Table 1, Supplementary Fig. 1). Table 1 shows that sIPSC amplitude in the presence of nicotine alone (21.3 ± 1.5 pA) was not significantly different from the amplitude in the presence of nicotine+WY+MK (25.1 ± 5.4 pA; P>0.05, n=7, paired t-test) or nicotine+Fenofibrate+MK ( 22.8 ± 1.9 pA; P>0.05, n=6, paired t-test).
